# Obesity Indices to Use for Identifying Metabolic Syndrome among Rural Adults in South Africa

**DOI:** 10.3390/ijerph17228321

**Published:** 2020-11-11

**Authors:** Mohlago A. Seloka, Moloko Matshipi, Peter M. Mphekgwana, Kotsedi D. Monyeki

**Affiliations:** 1 Department of Physiology and Environmental Health, University of Limpopo, Sovenga 0727, South Africa; mohlago9592@gmail.com (M.A.S.); kotsedi.monyeki@ul.ac.za (K.D.M.); 2 Research Administration and Development, University of Limpopo, Sovenga 0727, South Africa; peter.mphekgwana@ul.ac.za

**Keywords:** body mass index, confirmatory factor analysis, neck circumference, waist circumference, waist to height ratio

## Abstract

Metabolic syndrome (MetS) is a cluster of metabolic conditions that aggravate the likelihood of cardiovascular diseases and type 2 diabetes mellitus. This study was aimed to identify the best obesity index to determine MetS. This was a cross-sectional study and part of the Ellisras Longitudinal Study where 593 (289 males and 304 females) adults aged 22–30 years took part. Confirmatory factor analysis was used to test the single-factor models of MetS defined by mid arterial pressure, fasting blood glucose, triglycerides and commonly selected obesity indices such as Neck circumference (NC), Body mass index (BMI), Waist circumference (WC) and Waist to height ratio (WHtR) as indicators of MetS. It was found that a single model fit built based on WC and WHtR suggested a better fit index than NC and BMI in males, whereas, a model built on NC obtained a better fit index for females than other factor models. In conclusion, the result of the present study suggests that in rural Ellisras adult’s, WC and WHtR are the best obesity indices for determining MetS in males and NC in females than other indices. Hence, longitudinal studies are recommended to allow causality to be drawn between obesity indices and MetS.

## 1. Introduction

Metabolic syndrome (MetS) is a global health problem characterised by a cluster of metabolic conditions and is known to aggravate the likelihood of cardiovascular diseases (CDVs) and type 2 diabetes mellitus (DM2) [1,2]. According to the World Health Organisation (WHO) [3], each year, MetS accounts for 1.9 million deaths in low- and middle-income countries. In South Africa, the prevalence of MetS was stated to be 21.8%; with 15.6% being males and 24.8% females [4].

Discrepancies in the definition of MetS has been reported previously and is thought to arise from obesity indices which are considered a major risk factor [2,3,4,5]. Most definitions of MetS use Waist circumference (WC) as obesity index which is thought to differs by gender, ethnicity, region, population and age [1,2,3,4,5]. The joint interim statement consider gender and population in its criteria to diagnose MetS and is based on WC ≥ 94 cm for males and ≥ 80 cm for females together with other two risk factors of MetS such as hypertension, dyslipidaemia and DM2 [2].

Accumulating evidence indicates that the location and distribution of fat in the body can be a good indicator of MetS and related risk factors [6]. Therefore, it is of utmost importance to find out a reliable obesity/anthropometric index to determine overall obesity/body fat and visceral/abdominal obesity [7,8]. Obesity indices such as Body mass index (BMI), WC, Waist to height ratio (WHtR) and Neck circumference (NC) are used widely in clinical and epidemiological studies [9,10]. However, these indices were reported to be the best determinants of metabolic health complications such as insulin resistance, hypertension, dyslipidaemia, CVDs and MetS [8,9,10].

Studies indicated that, although BMI can be a good indicator of overall obesity and known to correlate positively with metabolic conditions it cannot discriminate between fat mass and fat-free mass [8,9,10]. Waist circumference and WHtR are used as the simplest and alternative anthropometric indices that assess central/abdominal obesity and are a precise reflection of the visceral fats which is strongly linked to metabolic complications [10,11]. However, WC is also not suitable for use in obese individuals as its measurement is affected by fullness (meals) and respiration [8,9]. Thus, emerging evidence points NC as a relevant technique to measure fat distribution due to its simplicity and ability to identify measures of MetS [12]. Moreover, NC was found to correlate positively with BMI, WC, WHtR in identifying measures of MetS [12].

Confirmatory factor analysis (CFA) was first developed in all areas of psychology including educational research to evaluate the latent structure of test instrument such as a questionnaire [13]. The CFA is applied mostly when developing new measures and construct validity; evaluation of new and existing measures; to examine the model effect and to test measurements invariance across groups or population [14]. Its main objective is to determine the relationship between indicators/observed variables and unobserved/latent factors [14].

Several studies have indicated that obesity indices can be used as a diagnostic tool to determine MetS, however, these were conducted mostly in developed countries [7,8,9,10,11,12,13,14,15]. The value of the previous obesity-based studies in a rural setting of South Africa is limited, the focus was more on finding the cut-off values of obesity indices to determine MetS and most were done in urban settings [16,17]. In the Ellisras population, it was only the relationship between blood pressure and anthropometric indicators in rural South African children that has been investigated thus far [18]. Cardiovascular risk factors constitute a significant health problem in Ellisras population [19], hence researching about these indices could help screen and diagnose MetS at an early stage and their use should be the priority in reducing and preventing complications associated with CVDs where MetS play a major role. The main aim of the study was to identify the best obesity index, which can be used to determine MetS among Ellisras rural adults aged 22–30 years. The objectives of the study were (1) to find the correlation between obesity indices and MetS components; (2) to evaluate the single-factor models of MetS defined by Mid-arterial pressure (MAP), Fasting blood glucose (FBG), Triglycerides (TG) and commonly selected obesity indices as indicators of MetS.

## 2. Materials and Methods

### 2.1. Geographical Region

Ellisras, which is currently known as Lephalale is a deeply rural area in Limpopo Province, South Africa, located adjacent to Botswana borders. Ellisras has 42 settlements with about 50,000 people dwelling there [20].

### 2.2. Sample

The study was cross-sectional and comprising of a total of 593 rural adults (289 males and 304 females) aged 22–30 years. The information regarding the ELS such as a detailed study design and procedure are reported elsewhere [21].

Using STATA, (StataCorp Lp, College Station, TX, USA) the sample size required for the study was calculated based on a power of 90% and a two tailed significance level of 5%. The prevalence of MetS 21.8% amongst South African [4] with the alternative proposed proportion of 30%. A total of 345 participants were required per sample size of the study site and a total of 593 participated in the current study [4]. The exclusion criteria included participants who were pregnant or lactating, did not fast before blood collection, with missing variables and lastly those on diabetes or hypertension medication. The study was approved by the Turfloop Research Ethics Committee (TREC) of the University of Limpopo (Project Identification ID: MREC/P/204/2013:IR) before the survey. Participants read and signed informed consent before participation.

### 2.3. Anthropometric Measurements

#### 2.3.1. Waist Circumference

Anthropometric measurements were conducted by a trained and experienced field worker on all the study participants, according to a standard procedure of the International Society for the Advancement of Kinanthropometry (ISAK) [22]. The Waist circumference (WC) was measured using a flexible steel tape (Delta surgical SA (PTY) Ltd., Johannesburg, Gauteng, South Africa) to the nearest 0.1 cm, while participants were in light clothing and a standing position [23]. The measurements were taken at the middle point between the lowest rib and iliac crest at the end of each gentle expiration [15]. The cut-off points of WC are WC ≥ 94 cm for males and ≥80 cm for females [2].

#### 2.3.2. Neck Circumference

Neck circumference (NC) was measured using a flexible tape (Delta surgical SA (PTY) Ltd., Johannesburg, Gauteng, South Africa) with the position of the head in a Frankfurt horizontal plane [24]. The participants were in a standing position and the measurements were taken to the nearest 0.1 cm. The tape was placed perpendicular to the long axis of the neck and around the inferior margin of the laryngeal prominence [24]. Neck circumference was measured just below the Adams’ apple in men having large Adam’s apple [25]. The cut-off points of NC are ≥37 cm for men and ≥34 cm for women [9].

#### 2.3.3. Body Mass Index (Body Weight)

Weight was measured using an electronic scale (Delta surgical SA (PTY) Ltd., Johannesburg, Gauteng, South Africa) to the nearest 0.1 kg and a Martin anthropometer was used to measure height to the nearest 0.1 cm [23]. The participant was measured in a standing position, in light clothing and without shoes [23]. The individuals with BMI values of below 18.5 kg/m^2^ were classified as underweight, BMI of 18.5–24.9 kg/m^2^ classified as normal weight, BMI of 25–29.9 kg/m^2^ classified as overweight and BMI of 30.0 kg/m^2^ and above as obese [26].

### 2.4. Blood Pressure

Systolic blood pressure (SBP) and Diastolic blood pressure (DBP) readings were taken after 5 min of rest, using an Omron electronic micronta monitoring kit [27]. The participants were seated with feet on the floor and the measurements were taken 3 times at a 5 minutes’ interval. The average of three blood pressure (SBP and DBP) readings were calculated. A blood pressure of 120/80 mmHg was considered normal and that of ≥130/85 mmHg was classified as hypertension [1]. Mean arterial pressure (MAP) is the average of SBP and DBP influenced by cardiac output and systematic vascular resistance throughout one cardiac cycle [28]. Mean arterial pressure is an indication of blood pressure and was calculated using the following equation [5,28].

MAP = DBP + 1/3 × (SBP − DBP)(1)

### 2.5. Blood Sample Collection and Processing

Participants were asked to fast for 8–10 h before blood collection in the morning by registered nurses from the Witpoort Hospital. The blood samples were immediately placed in a cool box containing ice after collection. The samples were then centrifuged for 15 min to obtain plasma and serum at 2500 rpm and placed in a freezer at a temperature of −80 °C for later analysis. Haemolysed and clotted samples were discarded. Blood analyses were done at the Medical Science Unit of the Department of Pathology and Medical Science of the University of Limpopo.

Beckman LX20^®^ Auto Analyser (Beckman Coulter Inc., Brea, CA, USA) was used to measure the plasma glucose using an enzymatic method that uses the glucose oxidase. High-density lipoprotein-cholesterol was measured using a unique detergent that solubilises only the HDL-C particles and releases HDL-C. Triglycerides levels were measured using enzymatic (lipase, glycerol kinase, glycerophosphate oxidase and horseradish peroxidase) spectrophotometric technique. All plasma lipid measurements were done following the AU480 Chemistry System from Beckman Coulter (Brea, CA, USA). The AU480 instrument was calibrated according to standard procedures. All measurements were done three times and the percentage of the coefficient of variation (% CV) was calculated. Measurements were repeated when the CV > 5%.

### 2.6. Quality Control

The standard procedure of the International Society for the Advancement of Kinanthropometry (ISAK), was used as a standard procedure for all training of anthropometric measurement of participants [22]. The reliability and validity of the anthropometric measurements are reported elsewhere [23,24,25,26,27,28,29,30]. Field workers undertook reliability testing as part of their training [23]. They were also trained to have a technical error of measurement (%TEM) for anthropometric variables that are within acceptable standards as per the ISAK.

### 2.7. Statistical Analyses

All the continuous variables were assessed for normality using the Shapiro–Wilk test and represented by mean and SD unless otherwise specified. The characteristics of the population were compared between males and females using independent *t*-test. Systolic blood pressure (SBP), Diastolic blood pressure (DBP), Waist circumference (WC), Body mass index (BMI), High density- lipoprotein-cholesterol (HDL-C), Fasting blood glucose (FBG) and Mean arterial pressure (MAP) were not normally distributed hence, log transformation was carried out for both sexes and used in subsequent analyses. The Pearson correlation coefficient was used to find the association between obesity indices and components of Metabolic syndrome (MetS). Confirmatory factor analysis (CFA) was used to test the single-factor models of MetS defined by MAP, FBG, TG and commonly selected obesity indices such as NC, BMI, WC and WHtR as indicators of MetS [31]. The model was reported on the standardised regression weight (standardised factor loading). We built a single-factor model of MetS similar to the study by Motamed and colleagues which was differentiated from each other by four obesity indices of Waist to height ratio WHR, WC, WHtR and BMI with four hypothesized single factor models [5]. However, in this study, we used NC as an obesity index instead of WHR. The MetS variable was treated as a latent variable (the variable that that is inferred, not directly observed, from other variables that are observed) [32].

Chi-square test with different fit indices, including Comparative fit index (CFI), Goodness-of-fit index (GFI), Akaike’s information criterion (AIC), Tucker Lewis index (TLI) and Root mean square error of approximation (RMSEA) were used to assess the models. A model with RMSEA < 0.06, CFI > 0.95, and TLI > 0.95 was regarded as a good model-data fit [33]. The model with the lowest AIC value was considered the best model [34]. A maximum likelihood estimation was used to analyses the covariance of the variables. All statistical analyses were performed using Stata 15 software (StataCorp LP., College Station, TX, USA).

## 3. Results

The characteristics of the participants are described in Table 1. The number of participants was stratified by sex, were 51% females and 49% males. In the general population, “only FBG (*p* < 0.371) was not statistically significant” while SBP, MAP, NC, BMI, WC, WHtR, HDL-C and TG were all statistically significant (*p* < 0.001) and DBP (*p* < 0.013). No significant difference was found between the mean FBG (*p* < 0.371) of males and females. Men had significantly (*p* < 0.001) higher SBP, MAP, NC, WHtR, and HDL values than women, while BMI, WC and TG were significantly (*p* < 0.001) higher in the general population of women.

Table 2 shows the correlation between MetS and obesity indices. There was a significant positive correlation between MAP and all obesity indices while no significant correlation was detected between FBG and obesity indices. On the other hand, a significant negative correlation was detected between HDL, BMI and WC. The correlation of BMI with WC (*r* = 0.870) is more than the correlation of BMI with NC (*r* = 0.182).

Table 3 demonstrates standardised factor loading values for each of the four observed variables per model. The *p*-values for the chi-square test were greater than 0.05 for all the models, suggesting that all the models are significant. The chi-square statistic obtained from CFA is sensitive to sample size and we, therefore, rely on other criteria such as CFI, TLI, RMSEA and AIC to determine how well a model matches the data. In males, all the single-factor models had RMSEA values closer to 0, CFI and TLI values greater than 0.95, indicating good fit indices, however, single model fit built based on WC and WHtR had the smallest AIC values (−2680 and −2662 respectively), suggesting better fit indices than NC and BMI. Single model fit built on NC obtained a better fit index (CFI = 0.90, TLI = 0.71, RMSEA = 0.05 and AIC = −429.21) for females.

## 4. Discussion

The study was aimed to identify the best obesity index, which can be used to determine Metabolic syndrome (MetS) among Ellisras rural adults. The main findings of the present study demonstrated a significant gender difference in obesity indices. It was found that Waist circumference (WC) and Waist to height ratio (WHtR) were the best indices in determining MetS in males than Neck circumference (NC) and Body mass index (BMI); while in females, NC was found to be the best determinants than WC, BMI and WHtR. This disagreed with the results by Gómez-Marcos and colleagues where it was found that BMI was the best determinant of MetS in males and WC in females [35]. However, the probable reason to account for these differences is that the participants were from a Spanish population and the study focused on a full adult age range (20–80 years). Furthermore, emerging evidence has pointed NC to be a reliable, relevant and best obesity index in females to determine MetS than other indices since it can be used to overcome limitations of WC, WHtR and BMI in circumstances such as in overweight or obese females, pregnant females or not, as well as in postmenopausal or menopausal status [36,37]. This is appropriate given that females in the current study are at childbearing age and ought to gain weight with each pregnancy.

According to, Zhang and colleagues gender difference among obesity indices are uncertain, but it was indicated that sex hormones, age, metabolism, anatomy and physiology might play a partial role in the clarification of this [38]. Hormones such as testosterone influences muscle mass to fat mass ratio in males where most develop android fat distribution mainly in the abdomen, chest and shoulder resulting with higher WC and WHtR [39]. Nonetheless, oestrogen influences fat distribution in females where most develop gynoid fat distribution mainly around the hips, thighs and bottom when ageing [39]. It has been found that excessive or expansion of fat in the abdomen or adipose tissue disrupts the distribution of fat and induces the release of free fatty acid and produces adipokines [40,41]. Short-term exposure to these free- fatty acids is associated with the release of insulin while long term exposure results in lipotoxicity [42]. However, this results in the insulin resistance of the muscle and the liver caused by reduced insulin production by the pancreatic β-cell [42] giving rise to metabolic complications. Body mass index in both males and females was not the best indicator of MetS, similar findings were reported by [5,6,7,8,9,10,11,12,13,14,15,16,17,18,19,20,21,22,23,24,25,26,27,28,29,30,31,32,33,34,35,36,37,38,39,40,41,42,43]. Several studies have indicated that, although BMI is extensively used in a population to assess overall body weight it has limitations that it cannot differentiate between fat mass and fat-free mass [10,11,12,13,14,15,16,17,18,19,20,21,22,23,24,25], which is as well found in the current study.

In the general population of Ellisras, WC, WHtR and NC were the best indices to determine MetS than BMI. Similar results were reported by [15,16,17,18,19,20,21,22,23,24,25,26,27,28,29,30,31,32,33,34,35,36,37,38,39,40,41,42,43] although, the studies used different sample size, had different ethnicity and used different variables in the methodology. The use of obesity indices in these population can help screen and diagnose MetS as well as identify individuals at risk of health complications. However, intervention programmes may be developed to help curb health complications associated with MetS by preventing and treating them while still at the early stages.

Future work is needed to build on the current studies’ results to investigate further on inflammatory variables such as C-reactive protein since it was proposed as a component of MetS, Homeostatic Model Assessment of Insulin Resistance (HOMA-IR) can be added on the model as a measure of insulin resistance [35] and lastly optimal cut-points of the obesity indices using Receiver operating characteristic (ROC) can be determined.

This was a cross-sectional study and did not allow cause and effect relationship to be drawn between obesity indices and MetS, hence longitudinal studies are recommended. The study focused on rural black adults; therefore, this should be investigated further on other ethnic groups living in an urban area to allow comparison and prevent generalisation since MetS differ by region, gender, ethnic group, and the criterion used to define it [40]. The study was conducted on a young adults population thus, these can be explored on different age groups among gender.

The strength of the current research was to the authors best knowledge, that this was the first study constructed on a single model based on four obesity indices to determine MetS in a rural black population in South Africa.

## 5. Conclusions

WC and WHtR were the best determinants of MetS in males and NC in females. The results of the current study suggest that obesity indices such as WC, WHtR and NC can be used as important diagnostic tools in determining MetS because of their ability to precisely reflect visceral fats which are strongly linked to metabolic complications.

## Figures and Tables

**Table 1:** Characteristics of the study participants presented as mean and SD.

Variable	Total (*n* = 593) Mean (SD)	Male (*n* = 289) Mean (SD)	Female (*n* = 304) Mean (SD)	*p*-Value
Age (years)	25.0 (1.95)	25.0 (1.92)	25.0 (1.97)	0.142
DBP (mm/Hg)	1.84 (0.06)	1.85 (0.06)	1.84 (0.06)	0.013 *
SBP (mm/Hg)	2.08 (0.05)	2.10 (0.04)	2.06 (0.04)	<0.001 **
MAP (mm/Hg)	1.93 (0.05)	1.95 (0.05)	1.92 (0.5)	<0.001 **
FBG (mg/dL)	0.73 (0.7)	5.45 (0.87)	5.52 (0.92)	0.371
NC (cm)	33.45 (3.00)	35.2 (42.44)	31.75 (2.44)	<0.001 **
BMI (kg/m^2^)	1.36 (0.93)	1.32 (0.64)	1.40 (0.10)	<0.001 **
WC (cm)	1.89 (0.07)	1.87 (0.05)	1.91 (0.08)	<0.001 **
WHtR (cm)	0.46 (0.08)	1.04 (0.60)	0.95 (0.51)	<0.001 **
HDL-C (mg/dL)	0.43 (0.12)	0.64 (0.12)	0.02 (0.19)	<0.001 **
TG (mg/dL)	0.99 (0.55)	0.43 (0.05)	0.50 (0.09)	<0.001 **

Log transformed variables, Shapiro–Wilk test, independent *t*-test, BMI = Body mass index, DBP = Diastolic blood pressure, SBP = Systolic blood pressure, FBG = Fasting blood glucose, HDL-C = High density lipoprotein-cholesterol, MAP = Mean arterial pressure, NC = Neck circumference, TG = Triglycerides, WHtR = Waist to height ratio, WC = Waist circumference, SD = standard deviation, * *p* < 0.05; ** *p* < 0.001.

**Table 2:** Pearson correlation coefficients for the association of obesity indices and components of MetS.

Variables	MAP mm/Hg	FBG (mg/L)	NC (cm)	BMI (kg/m^2^)	WC (cm)	WHtR (cm)	HDL-C (mg/L)
MAP (mm/Hg)	1						
FBG (mg/L)	0.115 **	1					
NC (cm)	0.317 **	0.018	1				
BMI (kg/m^2^)	0.086 *	0.046	0.182 *	1			
WC (cm)	0.106 **	0.048	0.283 **	0.870 **	1		
WHtR (cm)	0.019 *	0.053	0.101 *	0.895 **	0.954 **	1	
HDL-C (mg/L)	0.125 **	−0.096	0.013	−0.146 **	−0.107 **	−0.129 **	1

MAP = Mean arterial blood pressure, FBG = Fasting blood glucose, NC = Neck circumference, BMI = Body mass index. WC = Waist circumference, WHtR = Waist to height ratio, HDL-C = High density lipoprotein-cholesterol, TG = Triglyceride, * *p* < 0.05, ** *p* < 0.001.

**Table 3:** Goodness-of-fit statistics for various factor models of MetS stratified by sex.

Model of Fit Index for Males							Model of Fit Index for Females						
Factor Models	Estimates	Chi-Square (df)	CFI	TLI	RMSEA (CI) *p*-Value	AIC	Factor Models	Estimates	Chi-Square (df)	CFI	TLI	RMSEA (CI) *p*-Value	AIC
MAP	0.5273	Chi-square = 0.12 Df = 2 *p*-value = 0.9418	1.000	1.112	0.000 (0.000; 0.023) 0.971	−453.084	MAP	1.0000	Chi-square = 3.84 Df = 2 *p*-value = 0.1463	0.90	0.71	0.050 (0.000; 0.138) 0.352	−429.21
FBG	0.2227						FBG	0.1578					
TG	0.4786						TG	0.0952					
NC	0.4920						NC	0.1739					
MAP	0.4170	Chi-square = 1.05 Df = 2 *p*-value = 0.5905	1.000	1.056	120.000 (0.000; 0.097) 0.764	−2555.232	MAP	0.4787	Chi-square = 10.87 Df = 2 *p*-value = 0.0044	0.757	0.270	0.121 (0.058; 0.195) 0.035	−2373.84
FBG	0.2033						FBG	0.0949					
TG	0.6015						TG	0.2507					
BMI	0.4760						BMI	0.5534					
MAP	0.4208	Chi-square= 1.14 Df = 2 *p*-value = 0.5652	1.000	1.051	0.000 (0.000; 0.099) 0.747	−2680.055	MAP	0.3543	Chi-square= 11.36 Df = 2 *p*-value = 0.0034	0.696	0.089	0.124 (0.061; 0.198) 0.029	−2549.747
FBG	0.2254						FBG	0.0333					
TG	0.5824						TG	0.2938					
WC	0.4841						WC	0.6022					
MAP	0.4171	Chi-square= 1.32 Df = 2 *p*-value = 0.5178	1.000	1.046	0.000 (0.000; 0.103) 0.712	−2661.463	MAP	0.3055	Chi-square = 11.32 Df = 2 *p*-value = 0.0035	0.657	0.028	0.124 (0.061; 0.198) 0.030	−2533.703
FBG	0.2245						FBG	0.0188					
TG	0.5870						TG	0.3261					
WHtR	0.4312						WHtR	0.5746					

Chi-square test, MAP = Mean arterial pressure, FBG = Fasting blood glucose, NC = Neck circumference, BMI = Body mass index. WC = Waist circumference, WHtR = Waist to height ratio, HDL = High density lipoprotein, TG = Triglyceride, CFI = Comparative fit index, TLI = Tucker lewis index, RMSEA = Root mean square error of approximation, AIC = Akaike’s information criterion, DF = Degree of freedom.
